# Imaging and Clinical Features of Primary Thoracic Lymphangioma

**DOI:** 10.2174/0115734056346925241226125948

**Published:** 2025-01-03

**Authors:** Mingxia Zhang, Ling Li, Meng Huo, Lei Sun, Chunyan Zhang, Ying Sun, Rengui Wang

**Affiliations:** 1 Department of Radiology, Beijing Shijitan Hospital, Capital Medical University, Beijing, China

**Keywords:** Thoracic lymphangioma, Chest CT, Imaging features, Clinical manifestations, Clinical symptoms, GLA, D-dimer

## Abstract

**Background:**

Primary thoracic lymphangioma is a rare disease. Most of the previous studies are comprised of individual case reports, with a very limited number of patients included.

**Objective:**

This study aims to investigate the chest computed tomography (CT) imaging features and clinical manifestations of thoracic lymphangioma, thereby enhancing our understanding of the condition.

**Methods:**

A retrospective analysis was conducted on 62 patients diagnosed with thoracic lymphangioma, comprising 32 males and 30 females. The study focused on analyzing the chest CT imaging features and the clinical manifestations observed in these patients.

**Results:**

The incidence rates of thoracic lymphangioma did not differ significantly between males and females; however, it was more frequently observed in children and adolescents. The most common clinical symptoms included cough, fever, chylothorax, chylous pericardium, and lymphedema. The mediastinum (82.3%) emerged as the most frequent location for thoracic lymphangioma, followed by the chest wall (62.9%), bone (40.3%), and pleura (32.3%). Pulmonary lymphangioma, the least prevalent subtype (19.4%), exhibited a propensity to induce respiratory symptoms, frequently manifesting as a generalized lymphatic anomaly (GLA). Furthermore, elevated levels of D-dimer were detected in 34 patients (54.8%) with thoracic lymphangioma.

**Conclusion:**

Imaging examinations play a crucial role in assisting clinicians in making more accurate early diagnoses of thoracic lymphangioma. They are also helpful for assessing the extent of systemic infiltration and enhancing diagnostic precision. With radiological assessment, clinicians could more readily select appropriate therapeutic treatments and monitor the progression of follow-up care.

## 1 INTRODUCTION

Lymphangioma, predominantly a benign neoplasm, is a rare form of lymphangioma. While it is most frequently observed in infants and young children, it can also manifest in adults. Lymphangioma, which originates from lymphatic vessels, has the potential to manifest in any anatomical region harboring these structures, especially in the head and neck [1, 2]. Intrathoracic lymphangioma, a relatively uncommon subtype, has the potential to affect the lungs, mediastinum, pericardium, pleura, and chest wall [1]. The clinical manifestations of lymphangioma are often nonspecific, varying based on the site of involvement and the adjacent organs affected. When the mediastinum lungs, chest wall, and pleura are infiltrated by Lymphangioma, symptoms may include moderate dyspnea, dry cough, chest pain, chest pressure, and shortness of breath, with respiratory failure being a possible complication in severe cases.

Diagnosis of lymphangioma is challenging and relies on pathological analysis. Currently, there is no definitive and effective treatment, and the prognosis is contingent upon the severity of the disease and the involvement of vital organs. Patients with early-onset lymphangioma often experience a poorer prognosis [1]]. The majority of literature on this topic consists of case reports, with limited research available on its clinical and imaging features. The 11th workshop of the International Society for the Study of Vascular Anomalies (lSSVA) introduced the first classification system in 1996, which was subsequently updated at the 20th workshop in 2014. Common (cystic) lymphatic malformations (LMs), which include macrocy/stic, mlcrocvstic or mixed types, represent the most prevalent lymphatic abnormality in infants. These lesions typically present as isolated cystics of tissue masses, commonly found in the neck, mediastinum, and retroperitoneum [3].

Thoracic lymphangioma often coexists with multiple body parts, known as Generalized Lymphatic Anomaly (GLA). GLA is a rare multi-organ disorder that affects the lungs, mediastinum, spleen, and other organs, characterized by diffuse lymphatic proliferation. Chest involvement in GLA can lead to respiratory failure and is associated with a poor prognosis [4-6]. This condition primarily affects children and adolescents, presenting a range of symptoms. Gorham-Stout disease (GSD) is another rare condition, marked by the presence of intraosseous LM. As LMs develop and proliferate, osteolysis occurs in a destructive manner [7].

Although pathology is considered the “gold-standard” in clinical practice, the diversity of GLA and GSD lesions often precludes surgical intervention or biopsy in certain patients, who are also at risk of postoperative lymphatic leakage. Computed Tomography (CT) and Magnetic Resonance Imaging (MRI) serve as non-invasive diagnostic tools for lymphangiomatosis, effectively visualizing fluid-filled masses across various sites and organs [8-10]. Consequently, clinicians can establish a diagnosis by integrating clinical manifestations with Direct Lymphangiography (DLG) and CT lymphangiography (CTL).

In this study, 10 cases of localized cystic lymphangioma were pathologically verified through surgical resection, while 31 cases of GLA and 2 cases of GSD were confirmed pathologically. Additionally, 21 cases were diagnosed using a combination of clinical and imaging assessments, including radionuclide lymphoscintigraphy, MR thoracic duct imaging, DLG, and CTL.

## 2 METHODS

### 2.1 Study Population

Patients diagnosed with thoracic lymphangioma at Beijing Shijitan Hospital Affiliated with Capital Medical University between March 2015 and August 2023 were enrolled in this study. Inclusion criteria: (1) Lymphangioma involving the chest was diagnosed by clinical, radiological, or histological findings. Exclusion criteria: Patients with other underlying diseases and secondary causes of lymphatic obstruction were excluded. Among the 50 cases of GLA and 2 cases of GSD, 31 cases were diagnosed histopathologically. The remaining 21 cases were diagnosed by clinical and imaging assessments, including radionuclide lymphoscintigraphy, MR thoracic duct imaging, DLG, and CTL. All subjects gave their informed consent for inclusion before they participated in the study. The study was conducted in accordance with the Helsinki Declaration, and the protocol was approved by the Ethics Committee of Beijing Shijitan Hospital, Capital Medical University (Project identification code: IIT2024-059-001). The primary clinical characteristics of the 62 patients with thoracic lymphangioma are presented in Table **1**.

### 2.2 Examination Methods

All 62 patients underwent chest CT scans using a GE Revolution 256-row CT scanner, which covered the region from the lung apex to the base. The CT scanning parameters were set as follows: tube voltage 120 kV; collimator width 1mm; tube current 110 mAs. The raw data were reconstructed into images with a slice thickness of 2 mm and a slice interval of 2 mm, using a reconstruction matrix of 512 × 512.

Lymphatic surgery was conducted on patients with lymph node involvement. A total of 51 cases underwent DLG examination. Depending on the clinical assessment, Superficial lymphatic vessels were selected from the foot side (between the roots of the first to third toes), and these vessels were punctured. A volume of 6-20 ml of lipiodol was then injected at a rate of 6-8 ml/h. With the guidance of digital subtraction angiography (DSA), the development of the lymphatic vessels was dynamically monitored until the entrance to the thoracic duct was visualized.

A total of 34 patients underwent CTL, defined as CT scans performed within 30 minutes to 2 hours after DLG. All patients received non-contrast CT scans of the chest, abdomen, and pelvis, extending from the apex of the thoracic vertebrae to the pubic symphysis, using a GE Revolution 256-row CT scanner. The CT scanning parameters were set as described above.

### 2.3 Image Analysis

The chest CT image features of 62 cases were independently evaluated by two experienced radiologists. In cases of disagreement, the final decision was made by a third senior radiologist. The CT imaging features assessed included the location of thoracic lymphangioma (chest wall, pleura, mediastinum, lung, bone), morphological types [cystic (giant cystic, microcystic, mixed type)], imaging findings of pulmonary lymphangioma (reticular opacities, ground glass opacities, thickening of peribronchial vascular bundles), and massive osteolysis. Additionally, the presence of abnormal distribution and accumulation of contrast agents was determined in 34 cases undergoing CTL examination. The above findings were described in terms of frequency.

According to Giguere *et al*. [11], the classification of cystic lymphangioma lesions was based on the size of the cystic cavity. Cystic lesions were classified asmacrocystic when the cystic cavity measured greater than 2 cm. Conversely, if the cystic size was less than 2 cm, the lesion was considered microcystic. Lesions with both cystic sizes were subsequently categorized as mixed lesions.

## 3 RESULTS

### 3.1 Clinical Manifestations and Laboratory Indicators of Thoracic Lymphangioma

There was no significant difference in the incidence of thoracic lymphangioma between males and females. The clinical symptoms observed included cough, fever, chest tightness, breathlessness, hemoptysis, chylous sputum, chylothorax, chylpericardium, chest wall or axillary mass, neck mass, chest wall edema, skin vesicles, skin vermilous hyperplasia, and limb lymphedema. Laboratory tests revealed that D-dimer levels were abnormally elevated in 54.8% of patients (Table **1**).

### 3.2 Imaging Findings of Thoracic Lymphangioma

**Fig. (1):**
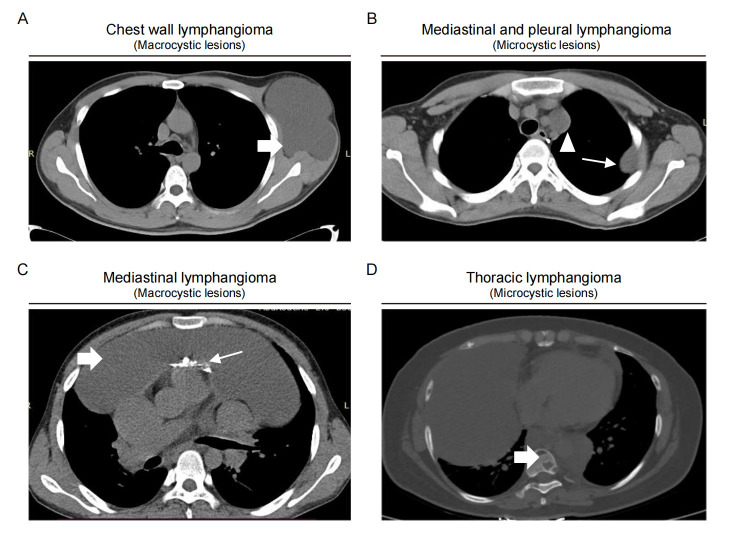
Imaging findings of thoracic lymphangioma. **A**. Chest wall lymphangioma, macrocystic lesions (thick white arrow). **B**. Mediastinal lymphangioma (white triangle) and pleural lymphangioma (thin white arrow), microcystic lesions. **C**. Mediastinal lymphangioma, macrocystic lesions (thick white arrow). Abnormally distributed contrast material (thin white arrow). **D**. Thoracic lymphangioma (thick white arrow), microcystic lesions.

The most common location for thoracic lymphangioma was the mediastinum, affecting 82.3% of cases, followed by the chest wall at 62.9%, bone at 40.3%, pleura at 32.3%. Pulmonary lymphangioma was the least common, found in 19.4% of cases. The mixed type of lymphangioma involving both the chest wall and mediastinum, was the most prevalent form. Meanwhile the microcystic type of pleural lymphan-gioma was the most common form. (Table **2** and Fig. **1**).

**Fig. (2):**
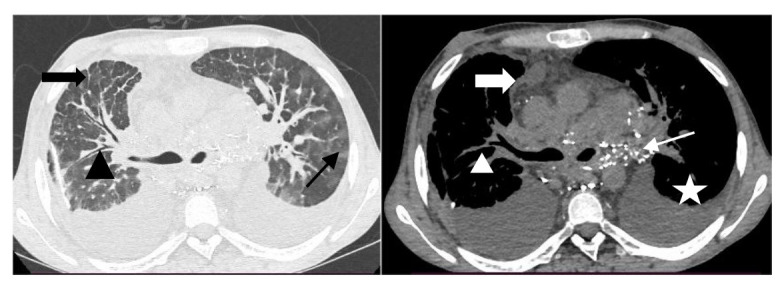
In one case of diffuse pulmonary lymphangioma, ground-glass opacities (thin black arrow), reticular opacities (thick black arrow), and thickening around the bronchovascular bundle (black triangle) were seen in the lung window (left). In the mediastinal window, there was thickening around the bronchovascular bundle of both lungs with low density (white triangle), accompanied by mediastinal lymphangioma (thick white arrow) and chylothorax (white five-pointed star). Abnormal distribution of contrast medium was also observed (thin white arrow) (right).

All 12 cases of pulmonary lymphangioma in this study exhibited diffuse pulmonary lymphangiomatosis (DPL), and each of the 12 cases demonstrated ground glass density shadows, reticular shadows, and thickening around the bronchovascular bundle simultaneously (Fig. **2**).

**Fig. (3):**
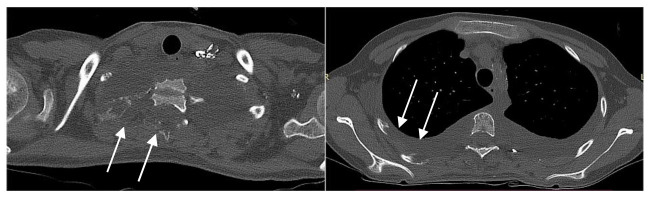
Massive osteolysis (thin white arrow) was seen in the vertebral body and ribs of one GSD patient.

Among thoracic and rib lymphangiomas, the majority presented cystic changes, with only two cases exhibiting massive osteolysis (Fig. **3**). Regarding CTL, 32 out of 34 patients (94.1%) with thoracic lymphangioma exhibited abnormal distribution and accumulation of the contrast agent.

## 4 DISCUSSION

In the present study, the 62 cases of thoracic lymphangioma exhibited no gender difference, aligning with prior research [12]. Thoracic lymphangioma typically presents with an insidious onset, and chylothorax emerged as the most common symptom among these 62 patients. The presence of chylothorax indicates an abnormality within the lymphatic system, highlighting the importance for clinicians to conduct chest CT examinations in such cases. Chest computed tomography (CT) examination is a rapid and facile diagnostic procedure. It facilitates the visual identification of fluid-like density masses within the chest wall, pleura, mediastinum, lungs, rib cage, as well as thoracic vertebrae. This imaging modality facilitates the precise determination of lesion size, number, and extent, thereby offering significant value in the initial assessment and diagnosis of various conditions. Furthermore, integrating chest CT with enhanced imaging techniques such as radionuclide lymphoscintigraphy, MR thoracic duct imaging, DLG, and CTL further enables accurate diagnosis by clinicians.

Symptoms of thoracic lymphangioma are often associated with the specific location of the affected lymphangioma. Localized cystic lymphangiomas may only manifest as a mass without additional symptoms, while those presenting solely as a chest wall or neck mass may have unsuspected low-density cystic lesions in the mediastinum and elsewhere, as revealed by chest CT. Given the disparate therapeutic approaches for localized cystic lymphangiomas and systemic lymphangiomas, routine chest CT screening is advised to prevent overlooking lymphangiomas in other thoracic regions. Symptoms such as chylothorax, chylouspericardium, chylous sputum, skin vesi-cles, and lymphedema of the chest wall and limbs indicate lymphatic system abnormalities. One case with skin verrucous hyperplasia exhibited lymphatic hyperplasia and dilation, and another case with skin vesicle resection showed lymphatic dilation, corroborating previous findings. In this study, DPL was characterized by cough, fever, and respiratory distress. Chest CT typically revealed low-density cystic disease in the mediastinum, particularly in the posterior mediastinum, accompanied by chylothorax. D-dimer levels were abnormally elevated in 54.8% of patients, predominantly in those with systemic lymphangioma. This finding suggests that patients with systemic lymphangioma may exhibit abnormal coagu-lation function, warranting further investigation.

The most frequent location of thoracic lymphangioma was the mediastinum (82.3%), followed by the chest wall (62.9%), bone (40.3%), pleura (32.3%), with lung lymphangioma being the least common (19.4%). The thoracic duct traverses the mediastinum, terminating at the jugular angle where it drains into the venoussystem [13]. DLG reveals that many patients with lymphangioma have thoracic duct outlet obstruction, frequently accompanied by lymphangiectasia and reflux at the proximal end of the obstruction. This observation may partially explain the high prevalence of mediastinal lymphangioma. The mixed type represented the most common form of lymphangioma impacting both the chest wall and mediastinum, whereas the microcystic type was the most prevalent among pleural lymphangiomas.

In this study, all 12 cases of pulmonary lymphangioma were diagnosed as DPL. All patients exhibited ground-glass opacity, reticular opacity, and peribronchial vascular bundle thickening concurrently. Although the remaining cases could also display these three imaging features in the lungs, they often did not present all three simultaneously. DPL is an exceptionally rare benign condition characterized by the abnormal proliferation, dilation, and thickening of lymphatic vessels within the soft tissues of the lung, pleura, and mediastinum. Imaging studies typically reveal thickening of the inter lobular septum and broncho vascular bundle, with the pathological basis being the aberrant proliferation and dilation of lymphatic vessels [14, 15].

In thoracic and rib lymphangiomas, the majority exhibited cystic changes, with only two cases demonstrating massive osteolysis. Rib involvement was observed in a single patient, while the rest had lymphangiomas involving multiple body parts. Cystic bone lymphangiomas appear as fluid-like low-density cystic shadows either round or oval in shape, with well-defined margins. The pathology is characterized by lymphatic proliferation and dilation. GSD is a rare condition with an unclear etiology, characterized by the presence of LM and attendant destructive osteolysis due to the growth and proliferation of LM. Typical CT findings include extensive bone loss, localized low-density areas, and discontinuous cortical bone [7, 16-18].

Among the 34 patients with thoracic lymphangioma who underwent CTL examination, 32 (94.1%) demonstrated abnormal distribution and accumulation of the contrast agent. This finding suggests a high prevalence of aberrant lymphatic return in patients with lymphangioma. It also means that clinicians should pay attention to the situation of abnormal lymphatic return in these patients and formulate personalized treatment plans based on the characteristics of different lymphatic return disorders, such as different obstruction sites and different types of lymphatic fistulas, to solve the problems associated with the abnormal lymphatic system, thereby achieving more effective treatment outcomes for lymphangioma.

This research acknowledges certain limitations. Firstly, the study is a single-center, small-sample investigation, lacking comparative analyses based on lymph node classification. Secondly, due to the limited number of CTL examinations, a detailed analysis of the abnormal distribution and accumulation of the contrast agent was not conducted, nor was a direct comparison made between CTL and DLG. Future efforts will focus on collecting additional cases to enhance the scope and depth of this study.

Applying multi-modal image segmentation methods for further analysis of thoracic lymphangioma can enhance diagnostic accuracy. Moreover, by comparing various AI image segmentation approaches, we can reduce image noise and improve image quality, thereby further increasing the diagnostic accuracy of thoracic lymphangioma. These methods will be adopted in our next-phase research [19, 20].

## CONCLUSION

In summary, thoracic lymphangioma is a rare disease characterized by non-specific clinical symptoms. Chest CT scanning offers a non-invasive, convenient, and rapid diagnostic tool, capable of swiftly and intuitively visualizing the location, size, and number of low-density cystic lesions associated with lymphangioma. Importantly, it can simultaneously depict ground-glass opacity, reticular opacities, and thickening of the bronchial vascular bundle, among other pulmonary interstitial changes. This enables an early determination of the type of thoracic lymphangioma. By leveraging CT imaging, in conjunction with MR thoracic duct imaging, DLG, and CTL, the risk of missed diagnosis and misdiagnosis can be minimized, thereby establishing a solid foundation for subsequent treatment planning and follow-up care.

## Figures and Tables

**Table 1:** Clinical manifestations and laboratory parameters of thoracic lymphangioma.

**Clinical Measures**	**Number of Cases**	**Proportion (%)**
Gender (Male)	32	51.6
Cough	11	17.7
Fever	9	14.5
Chest tightness and breathlessness	20	32.3
Hemoptysis	1	1.6
Chylous sputum	2	3.2
Chylothorax	24	38.7
Chylous pericardium	14	22.6
Chest wall and axillary mass	10	16.1
Neck mass	3	4.8
Chest wall edema	7	11.3
Skin vesicles	3	4.8
Verrucous hyperplasia of the skin	1	1.6
Lymphedema of the extremities	14	22.6
Chyle leakage	4	6.5
Asymptomatic based on physical examination	5	8.1
D-dimer increasing	34	54.8

**Table 2:** Imaging findings of thoracic lymphangioma.

**Imaging Findings**	**Number of Cases**	**Proportion (%)**
**Wall of chest**	39	62.9
Macrocystic lesions	6	9.7
Microcystic lesions	21	33.9
Mixed lesions	12	19.4
**Mediastinum**	51	82.3
Macrocystic lesions	7	11.3
Microcystic lesions	23	37.1
Mixed lesions	21	33.9
**The mediastinal lymph nodes were enlarged**	13	20.1
**Mediastinal emphysema**	3	4.8
**The pericardium was thickened**	33	53.2
**Pericardial effusion**	27	43.5
**Pleura**	20	32.3
Macrocystic lesions	1	1.6
Microcystic lesions	14	22.6
Mixed lesions	5	8.1
**The pleura was thickened**	30	48.4
**Pleural effusion**	28	45.2
**Pneumothorax**	2	3.2
**Lung**	12	19.4
**Pulmonary reticular shadow**	23	37.1
**Ground-glass opacities in the lungs**	15	24.2
**Thickening around the bronchovascular bundle**	18	29.0
**Ribs, thoracic vertebrae**	25	40.3
Macrocystic lesions	0	0
Microcystic lesions	19	30.6
Mixed lesions	4	6.5
Massive osteolysis	2	3.2

## Data Availability

The data and materials used in this study are available upon reasonable request by the corresponding author [R.W.].

## LIST OF ABBREVIATIONS

CT: Computed Tomography
GLA: Generalized Lymphatic Anomaly
ISSVA: International Society for the Study of Vascular Anomalies
LMs: Lymphatic Malformations
GSD: Gorham-Stout Disease
MRI: Magnetic Resonance Imaging
DLG: Direct Lymphangiography
CTL: Computed Tomography Lymphangiography
DSA: Digital Subtraction Angiography
DPL: Diffuse Pulmonary Lymphangiomatosis

## STANDARDS OF REPORTING

STROBE guidelines were followed.
